# Artificial Anisotropy in Ge_2_Sb_2_Te_5_ Thin Films after Femtosecond Laser Irradiation

**DOI:** 10.3390/ma15103499

**Published:** 2022-05-13

**Authors:** Aleksandr Kolchin, Dmitrii Shuleiko, Mikhail Martyshov, Aleksandra Efimova, Leonid Golovan, Denis Presnov, Tatiana Kunkel, Victoriia Glukhenkaya, Petr Lazarenko, Pavel Kashkarov, Stanislav Zabotnov, Sergey Kozyukhin

**Affiliations:** 1 Faculty of Physics, Lomonosov Moscow State University (MSU), 1/2 Leninskie Gory, 119991 Moscow, Russia; avkolchin@physics.msu.ru (A.K.); shuleyko.dmitriy@physics.msu.ru (D.S.); martyshov@physics.msu.ru (M.M.); efimova@vega.phys.msu.ru (A.E.); golovan@physics.msu.ru (L.G.); denis.presnov@phys.msu.ru (D.P.); kashkarov@physics.msu.ru (P.K.); 2 Skobeltsyn Institute of Nuclear Physics, Lomonosov Moscow State University (MSU), 1/2 Leninskie Gory, 119991 Moscow, Russia; 3 Quantum Technology Center, Lomonosov Moscow State University (MSU), 1/35 Leninskie Gory, 119991 Moscow, Russia; 4 Moscow Institute of Physics and Technology, 9 Institutskiy Per., 141701 Dolgoprudny, Russia; kunkel.ts@phystech.edu; 5 Kurnakov Institute of General and Inorganic Chemistry of the Russian Academy of Sciences, 31 Leninsky Avenue, 119991 Moscow, Russia; sergkoz@igic.ras.ru; 6 Institute of Advanced Materials and Technologies, National Research University of Electronic Technology, 1 Shokina Sq., 124498 Zelenograd, Russia; kapakycek2009@yandex.ru (V.G.); lpi@org.miet.ru (P.L.); 7 National Research Center “Kurchatov Institute”, 1 Akademika Kurchatova Square, 123182 Moscow, Russia

**Keywords:** GST225, femtosecond direct laser writing, Raman spectroscopy, conductivity anisotropy, infrared spectroscopy

## Abstract

Ge_2_Sb_2_Te_5_ (GST225) looks to be a promising material for rewritable memory devices due to its relatively easy processing and high optical and electrophysical contrast for the crystalline and amorphous phases. In the present work, we combined the possibilities of crystallization and anisotropic structures fabrication using femtosecond laser treatment at the 1250 nm wavelength of 200 nm thin amorphous GST225 films on silicon oxide/silicon substrates. A raster treatment mode and photoexcited surface plasmon polariton generation allowed us to produce mutually orthogonal periodic structures, such as scanline tracks (the period is 120 ± 10 μm) and laser-induced gratings (the period is 1100 ± 50 nm), respectively. Alternating crystalline and amorphous phases at the irradiated surfaces were revealed according to Raman spectroscopy and optical microscopy studies for both types of structures. Such periodic modulation leads to artificial optical and electrophysical anisotropy. Reflectance spectra in the near infrared range differ for various polarizations of probing light, and this mainly results from the presence of laser-induced periodic surface structures. On the other hand, the scanline tracks cause strong conductivity anisotropy for dc measurements in the temperature range of 200–400 K. The obtained results are promising for designing new GST225-based memory devices in which anisotropy may promote increasing the information recording density.

## 1. Introduction

Development of nanotechnologies allows for the fabrication of new semiconductor media and devices with desirable electronic and optical properties, including objects which possess artificial anisotropy. The latter is a well-known phenomenon explained by the preferable orientation of components with non-spherical symmetry inside a composite medium [1]. When applying dc or for the component size much less than an optical wavelength, these media could be characterized by effective dielectric function [2,3,4,5], which should take into account the anisotropy of component forms and distributions [6,7]. From a practical viewpoint, the so-called in-plane anisotropy with a strong difference of optical or electrical properties, which are determined by different directions of corresponding electromagnetic field vectors or currents applied along the sample surface, is of great interest. In-plane anisotropic structures can be formed by means of anisotropic chemical etching, as was demonstrated in experiments with mesoporous silicon [8,9,10,11]. Direct laser writing can also be applied to fabricate media with artificial anisotropy of surface relief [12,13,14,15,16,17]. Here, femtosecond lasers were used. They allow the fabrication of laser-induced periodic surface structures (LIPSS) of wavelength and sub-wavelength periods due to fast photoexcitation of surface plasmon polariton modes [18,19,20,21]. The surface relief anisotropy leads to the occurrence of strong in-plain optical [16] and conductivity [17] anisotropy. Periodic reliefs with a significant optical retardance can also be produced in the volume of different glasses with the help of femtosecond laser pulses [22,23,24], which opens new prospects for optical high-capacity information recording.

The employment of amorphous semiconductors should be noted, especially since, in this case, the anisotropy of the irradiated region can be caused, at least partially, by laser-induced periodical crystallization, as has been demonstrated in the experiments on femtosecond laser irradiation of amorphous silicon [16,17].

The formation of LIPSS also opens wide possibilities for rewritable optical memory. For this purpose, chalcogenide alloys, such as Ge_2_Sb_2_Te_5_ (GST225), may be applied. This material is well-known as a basic substance for non-volatile and rewritable memory applications, with a large difference between optical or electrical properties in the amorphous and crystalline states [25]. Recently, the fabrication of LIPSS oriented along or perpendicular to the laser polarization in thin GST255 films under femtosecond laser irradiation was demonstrated, including reversible phase transitions in corresponding regimes [26,27,28]. Moreover, prolate ellipsoidal crystalline particles nucleation was theoretically predicted at the crystallization of GST225-like systems in a strong laser field [29]; the ellipsoid axes are expected to be aligned along the direction of an applied laser radiation polarization. Both cases imply artificial optical anisotropy of the irradiated layers. However, conductivity and optical reflection anisotropy have not yet been experimentally studied in detail in such structures.

Herein, we fabricated a periodic relief in GST225 amorphous thin films via femtosecond laser irradiation in a regime of crystallization and LIPSS formation in the same sample. In-plane electrophysical anisotropy at different temperatures and anisotropy of reflectance in the near infrared range were examined for the irradiated samples and a comparison with the initial films was carried out.

## 2. Materials and Methods

Initial amorphous GST225 thin films with 200 ± 20 nm thickness were deposited by magnetron sputtering (MVU TM Magna 10) with a crystalline target (ACI Alloys) on preliminary thermally oxidized single-crystalline silicon substrates. The silicon oxide layer thickness was 1000 ± 40 nm. The Ar pressure and sputtering power in the chamber were 5.7 × 10^−1^ Pa and 25 W, respectively. The initial pressure in the chamber was 3 × 10^−3^ Pa. A detailed description of as-deposited sample characterization methods is given elsewhere [26].

Fabricated GST225 films were irradiated by femtosecond laser pulses in the air medium. Femtosecond laser system Avesta (central wavelength *λ* = 1.25 μm, repetition rate *ν* = 10 Hz, pulse duration *τ* = 135 fs, fluence *F* = 0.2 J/cm^2^) was used. The samples were moved by Standa automized mechanical translators in raster mode, while being irradiated at normal incidence by a linearly polarized laser beam focused by a lens with a focal length of 80 mm to a spot with the diameter *D* = 100 μm. The step between the raster scanlines *Γ* was equal to 120 μm to exclude possible intersections of neighbor scanlines and reamorphization effects within irradiated areas due to increased exposure time [28]. The scanning velocity was *V* = 5 μm/s. As a result, the square areas with the sizes 3 × 3 mm^2^ were formed. The pulse number per spot *N_s_* = 240 was defined by the formula [28]:

*N*_s_ = *ν*·*D*/*V* (1)

Images of as-deposited and irradiated surfaces were obtained by scanning electron (SEM; Carl Zeiss Supra 40, Carl Zeiss AG, Oberkochen, Germany) and optical microscopes (OM; Olympus BX41, Olympus Corporation, Tokyo, Japan). Phase composition of the as-deposited and irradiated films was studied via Raman spectra analysis (Horiba Jobin Yvon HR800; 488 nm) using a notch filter with the cut-off width of 100 cm^−1^. The accumulation time and the number of measurements were 40 s and 16, respectively.

The optical reflectance spectra of initial and irradiated samples in the visible and near-infrared (IR) range (0.8–2 μm) were measured using a Bruker IFS-66v/S IR Fourier-spectrometer for the incident angle α = 13° to the normal line. The measurements were carried out using unpolarized and polarized radiation. The number of measurements and spectral resolution were 30 and 1 cm^−1^, respectively.

**Figure 1:**
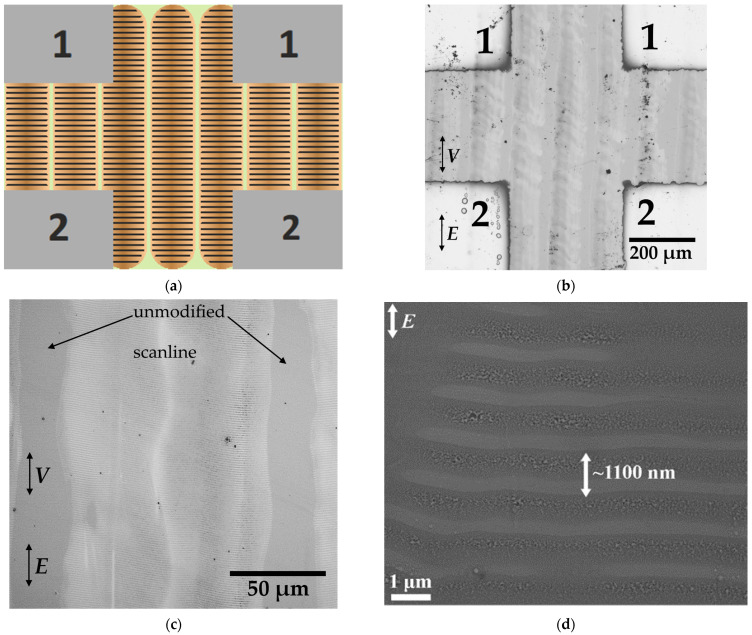
Scheme of the studied sample, where the gray areas illustrate the deposited aluminum electrodes for the electrophysical measurements; scanlines are depicted by vertical brown stripes, and the LIPSS within them are indicated by black horizontal shading (**a**). OM image of the fabricated sample with the deposited aluminum contacts (**b**); OM image (**c**) and SEM micrograph image (**d**) of irradiated surface of the sample. Numbers 1 and 2 in subfigures (**a**,**b**) mark electrodes for electrophysical measurements: the applied current may be flow between electrode pairs 1–1 or 1–2.

Electrophysical properties of the as-deposited and irradiated GST225 samples were studied using a Keithley 6487 picoampermeter (Keithley Instruments Inc., Singapore) and an ARS DE-204SE helium cryostat (Advanced Research Systems, Macungie, PA, USA). Four aluminum electrodes were deposited by thermal sputtering onto as-deposited and irradiated surfaces, providing the measurements in the mutually orthogonal directions in the surface plane. The distance between all the electrodes was the same and equaled to 340 μm. For the irradiated GST225 film, the direction of current flow between electrodes 1–1 was orthogonal to the laser beam scanlines, and the one between electrodes 1–2 was parallel to the scanlines, as shown in Figure 1a. Temperature dependencies of conductivity for the as-deposited and irradiated samples were measured in the range of 200–400 K, with 5 K step for 5 V direct voltage.

## 3. Results and Discussion

### 3.1. Structural Properties

The OM and SEM images of the irradiated samples given in Figure 1b–d demonstrate the presence of the scanline tracks with a width and period of *D* = 95 ± 10 μm and *Γ* = 120 ± 10 μm, correspondingly, as well as LIPSS with a period of *Λ* = 1100 ± 50 nm. The scanline parameters correspond to the ones from Section 2. The areas between the scanlines with the transversal size of 25 ± 10 μm appear as unmodified, and we will consider them as amorphous GST225 below. The formed LIPSS are directed orthogonally to the polarization of laser radiation. The mutual orientation of the scanlines and the LIPSS within them is orthogonal (Figure 1a).

The formed LIPSS may indicate surface plasmon polariton (SPP) generation during the intensive photoexcitation of the free charge carriers by high-power ultrashort laser pulses [28]. The necessary condition of SPP excitation is the metallization of the near-surface layer of the film under laser irradiation [18,19]. According to our previous estimations, the real part of GST225 dielectric function changes its sign from positive to negative at a carrier concentration of ~4 × 10^19^ cm^−3^ [28]. The density of charge carriers *n_e_* induced by a single laser pulse can be estimated from the total power, duration, and shape of a given pulse, taking into account the absorption and reflection coefficients of the irradiated GST225 film. According to a set of equations given elsewhere [28], the radiation parameters used in our work induce *n_e_ ≈* 6∙10^21^ cm^−3^, which exceeds the metallization threshold.

OM data (Figure 1c) show that irradiated areas within the scanlines contain alternating horizontal stripes of high and low reflectivity with period *Λ*. Considering the relatively low height of the LIPSS (less than 10 nm, according to our previous study [28]) and the much higher refractive index of crystallized GST225 [25] compared to its amorphous phase, we can associate stripes of high reflectance with the periodic crystallization of GST225 accompanying the formation of the LIPSS. The observed periodic distribution of the GST225 crystalline phase within the modified surface can be explained by the fact that the LIPSS are formed due to modulated film modification caused by a laser-induced stationary wave, which is emerging as a result of excited SPPs and incident radiation interference [18,19,20]. Such stationary wave possesses fluence insufficient for crystallization at its nodes; thus, the GST225 crystalline phase is formed only in the antinodes.

**Figure 2:**
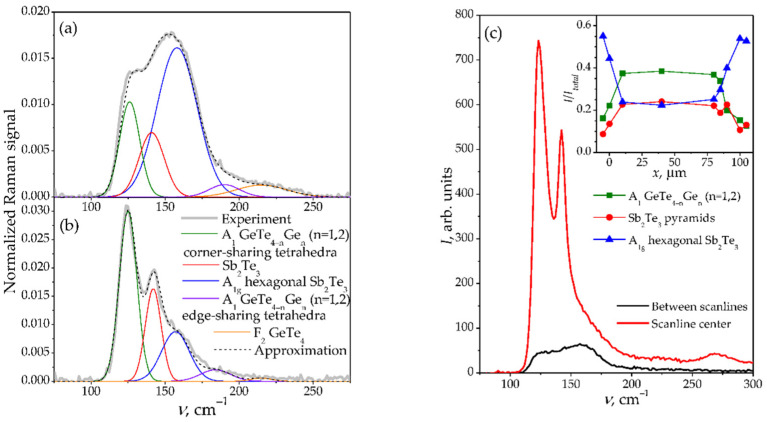
Raman spectra of as-deposited (**a**) and irradiated (**b**) GST225 samples, normalized by the integrated intensity of the whole spectra. Comparison of initial Raman spectra obtained between scanlines and at scanline center without normalization (**c**). The inset shows integrated intensities of Raman lines at 125 cm^−1^, 140 cm^−1^, and 158 cm^−1^ along the scanline cross section, normalized by the integrated intensity of the whole spectra. The diameter of the probing Raman beam equals 5 μm.

The crystallization of irradiated areas was confirmed by Raman spectroscopy data. As can be seen, the Raman spectrum of the initial film demonstrates an asymmetric broadband within the wavenumber *ν* range from 110 cm^−1^ to 250 cm^−1^ (Figure 2a), which is explained by the short-range order of the amorphous phase [30]. The form of this band can be approximated by 5 Gaussian lines peaking at 125 cm^−1^, 140 cm^−1^, 158 cm^−1^, 190 cm^−1^, and 215 cm^−1^, respectively. The 125 cm^−1^ and 190 cm^−1^ lines are associated with the A_1_ vibrational modes of the GeTe_4−n_Ge_n_ (n = 1,2) corner-sharing and edge-sharing tetrahedra, respectively [30,31]. The lines at 140 cm^−1^ and 158 cm^−1^ correspond to the Sb_2_Te_3_ pyramids mode [32] and the A_1g_^2^ mode of hexagonal Sb_2_Te_3_ [33], respectively. The line near 215 cm^−1^ is associated with the F_2_ mode of the GeTe_4_ vibrations [34].

After the femtosecond laser irradiation, the Raman spectrum at the center of the scanlines shows the next transformations (Figure 2b). The position of the lines remains practically unchanged. However, the integrated intensity of the 158 cm^−1^ line corresponding to the A_1g_^2^ mode of hexagonal Sb_2_Te_3_ decreases dramatically, as well as the 215 cm^−1^ line intensity. On the other hand, the intensities of the lines near 125 cm^−1^ and 140 cm^−1^ that correspond to the A_1_ modes of the GeTe_4−n_Ge_n_ (n = 1,2) corner-sharing and Sb_2_Te_3_ pyramids mode, increase in comparison to the rest of the spectrum. The intensity of the line at 190 cm^−1^ does not change significantly. Additionally, a small peak near 270 cm^−1^ was observed in the irradiated samples (Figure 2c), which may be ascribed to the Ge–Ge bonds [35]. To evaluate the uniformity of the laser-induced modification within the scanline, Raman spectra were obtained for several spots on the sample surface along the scanline cross section. The results of this Raman mapping are given in the inset of Figure 2c, indicating that within the scanline cross section, the intensity of the corresponding Raman lines, normalized by the whole spectrum intensity, does not change substantially. However, the Raman spectrum intensity itself for the irradiated GST225 film increases by ~6 times, compared to both the unmodified areas between the scanlines and the as-deposited film (Figure 2c), which indicates increased optical absorption, presumably due to film crystallization [36].

After the irradiation, some narrowing of the spectral lines is observed, which indicates an increase in the orderliness of the material. The full width at half maximum (FWHM) of the line at 125 cm^−1^ changes from 18 ± 2 to 13.3 ± 0.5 cm^−1^. The FWHM of the line at 140 cm^−1^ also decreases from 14.6 ± 1.2 to 12.0 ± 0.6 cm^−1^. Other spectral lines do not change FWHM significantly: it equals 34 ± 2 cm^−1^ for 158 cm^−1^, 24 ± 2 cm^−1^ for 190 cm^−1^, and 40 ± 4 cm^−1^ for 215 cm^−1^. The decrease in the intensity of the line at 158 cm^−1^, with a simultaneous increase in the intensity of the lines at 125 cm^−1^ and 140 cm^−1^, indicates that the phase transition presumably occurs mainly via the rearrangement of the GeTe structural units.

Thus, the observed Raman spectra transformation most likely indicates a transition from an amorphous to a face-centered cubic (fcc) crystalline structure [30,37] after laser treatment. The observed laser-induced crystallization of GST225 thin films can be described by the nucleation processes, with subsequent nanocrystal growth [38,39]. In particular, the forming nuclei may be needle-shaped crystal particles aligned along the applied laser field [29]. Therefore, the shape of these particles may additionally lead to the formation of the anisotropy [1] of the treated samples, as was shown previously in experiments with femtosecond laser irradiated amorphous silicon films [17].

### 3.2. Optical Properties

**Figure 3:**
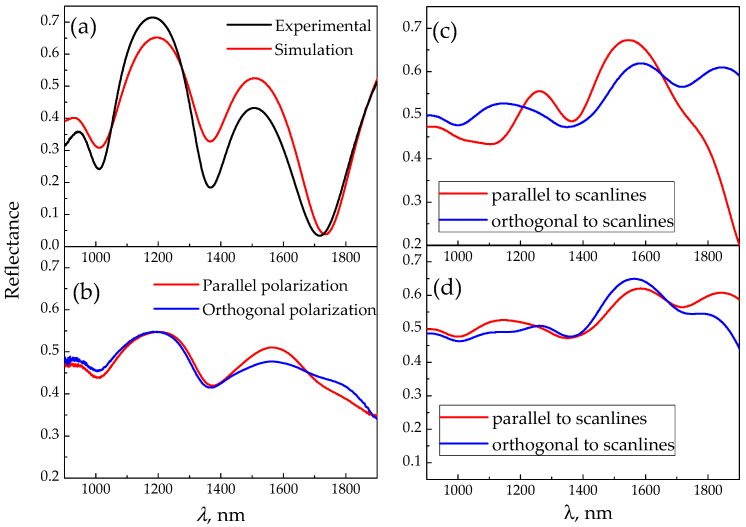
IR reflection spectra of as-deposited (unpolarized light) (**a**) and irradiated (**b**) GST225 samples. Calculated reflectance spectra for the scanlines considered as laminar structure caused by presence of LIPSS (**c**) and the array of nanocrystals oriented along laser radiation polarization in amorphous surrounding (**d**). The latter is characterized by a 50% volume fraction in both calculations. The ratio of ellipsoid semi-axes is equal to 10.

The measured reflectance spectra of the initial (Figure 3a) and irradiated (Figure 3b) GST225 samples show oscillations in the wavelength range of 800–2000 nm caused by the interference in the GST225 and SiO_2_ layers. Simulation of the reflectance spectrum was carried out by means of the transfer matrix method [1] for the GST225 and SiO_2_ layers on silicon substrate. For the calculations, the most suitable thicknesses of these layers were chosen as 210 nm and 970 nm, respectively, taking into account the dispersion of the real and imaginary part of the refractive indices in the spectral region [40,41]. The used thicknesses fall within the error band (confidence band) of the experimentally defined values (see Section 2). The simulation yields a reasonable agreement with the experimental spectrum for the initial sample of amorphous GST225 in the absence of anisotropy (Figure 3a).

The reflectance spectrum of the irradiated GST225 sample demonstrates a difference for the light polarization, both parallel and perpendicular to the scanlines in the region of 1400–1900 nm (Figure 3b). This effect could be ascribed to artificial optical anisotropy (birefringence and dichroism) in the irradiated GST225 regions caused by (i) the formation of a laminar structure by alternating crystalline and amorphous stripes perpendicular to the laser polarization, or (ii) the formation of nanocrystallites oriented along the laser polarization in the amorphous surrounding [29]. A contribution to the artificial anisotropy of large-scale components, such as the nonuniform scanline tracks and non-crystallized areas between them (Figure 1b–d), is not taken into account itself because the typical period of such components is on the order of 100 μm, and is much larger than the wavelengths within the spectral range under consideration. For such a scale ratio, we cannot use the approximation of the effective medium [2,3,4,5,6,7]. The composite optical reflectance should be defined as a sum of the contributions of the anisotropic partially crystallized layer, corresponding to the scanline tracks, and the amorphous non-modified layer, which corresponds to the areas between the scanlines. These contributions are taken in account with the weights of 0.79 and 0.21, respectively, in accordance with the transverse geometric sizes of these areas, mentioned in Section 3.1.

Since the relief height at the irradiated surface (about 10 nm, according to [28]) is two orders of magnitude less than a probing optical wavelength in the near IR range, we cannot expect a significant impact of scattering on the optical properties of the sample. To describe the optical properties, we chose to use the effective medium approximation. Simulation for both cases (i) and (ii) were carried out in the framework of the well-known generalized Bruggeman model [6,7] for a laminar structure and ellipsoids, respectively, including consideration of absorption as the refractive index imaginary part. The results are presented in Figure 3c,d. Although the employed simplified models do not yield a good quantitative agreement with the experimental data, they allows us to draw some qualitative conclusions. If the LIPSS within the scanlines is considered as a laminar structure, consisting of crystalline and amorphous stripes orthogonal to the modifying laser polarization, such structure possesses properties of a negative uniaxial crystal with an optical axis lying in the plane of the irradiated film normal to the LIPSS [1]. Thus, the optical axis is co-directed with the scanlines (Figure 1a). In this case, for a broad range of crystalline/amorphous volume fractions (25–75%) in the spectral region of 1400–1650 nm, reflectance for the radiation polarized orthogonal to the scanlines is less than that for the radiation polarized parallel to the scanlines, and vice versa in the region of 1650–1900 nm (see example in Figure 3c). This qualitatively agrees with the spectra found in the experiment. In contrast, for the case of prolate crystalline ellipsoids oriented along modifying laser polarization, the irradiated region has properties of a positive uniaxial crystal [6]; simulations for a broad range of the crystalline volume fractions (25–75%) and the ratios of ellipsoid semi-axes (2–100) evidence higher reflectance for the polarization parallel to the scanlines than the one for the polarization perpendicular to the scanline in the spectral region 1600–1900 nm (see example in Figure 3d), which is not supported by experimental results. Thus, the observed optical reflectance anisotropy of the irradiated GST225 surface is presumably caused by the presence of the LIPSS, which can be considered as amorphous and crystallized stripes, alternating with the period *Λ*.

### 3.3. Electrophysical Properties

**Figure 4:**
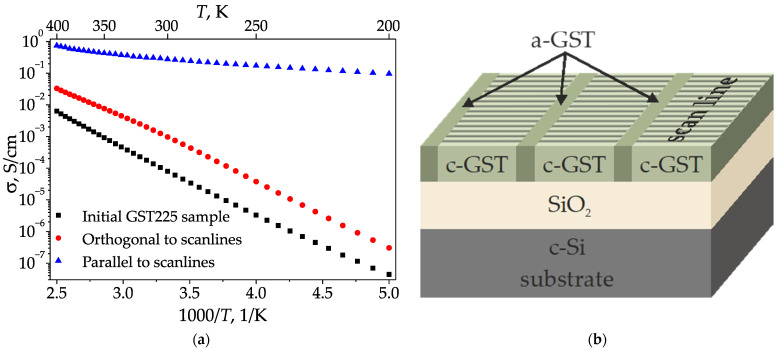
Temperature dependencies of conductivity (**a**) for the as-deposited sample and for the irradiated areas along and orthogonal to the scanlines. The scheme of crystalline and amorphous phases distribution inside the irradiated GST sample (**b**). Thin horizontal stripes indicate the presence of LIPSS within the scanlines; a-GST is amorphous GST225, c-Si and c-GST are crystalline silicon and crystalline GST225, respectively.

The temperature dependencies of the specific conductivity for the as-deposited and irradiated samples are given in Figure 4a. As can be seen, the conductivity value for amorphous GST225 increases from 4.4 × 10^−8^ S/cm to 6.3 × 10^−3^ S/cm during heating from 200 K to 400 K. A similar behavior was observed for irradiated samples in the direction 1–1 (Figure 1a), which is orthogonal to the scanlines. The conductivity in this direction also increases from 3.1 × 10^−7^ S/cm to 3.3 × 10^−2^ S/cm during heating in the same temperature range.

At the same time, in the direction 1–2, which is along the scanlines, the specific conductivity is 1–5 orders of magnitude higher, and changes from 9.6 × 10^−2^ S/cm to 0.74 S/cm with an increase in temperature from 200 K to 400 K.

The observed temperature dependencies of the specific conductivity *σ* are approximated by the formula:

(2) $$\sigma =\sigma_{0}\exp\left(-\frac{E_{a}}{kT}\right)$$

where *σ*_0_ is a fitting conductivity constant, *T* is the temperature, and *k* = 1.38 × 10^−23^ J/K is the Boltzmann constant. This allows us to define the activation energy value *E_a_* via the approximation of the line slope in the semi-logarithmic scale. The calculated value of the activation energy for the initial sample is 0.41 ± 0.01 eV, which agrees with the previous data [39]. The activation energy for the conductivity in direction 1–1 orthogonal to the scanlines is 0.40 ± 0.01 eV and does not differ dramatically from the activation energy of the unirradiated sample. Thus, we can assume that in this direction, the conductivity is determined mainly by the amorphous GST225 phase. At the same time, the activation energy along the scanlines (direction 1–2 in Figure 1a,b) is significantly lower and is equal to 0.06 ± 0.01 eV, which is close to the value for the crystalline fcc GST225 thin films [42] and is in a good agreement with the Raman analysis of this state (Section 3.1).

In the case of applying dc, a contribution of large-scale areas inside and outside of the scanline tracks should additionally be considered, because in such cases, dc may be considered as an electromagnetic wave, with the infinite wavelength when the effective medium approximation may be used to explain the artificial anisotropy. Actually, the high conductivity along the scanlines and the low conductivity in the perpendicular direction may be related to the presence of amorphous regions outside the scanlines (Figure 4b) [43]. According to this assumption, the charge carriers transport occurs preferentially along the scanlines in their central channels, where the maximum fluence upon irradiation leads to effective crystallization (see Section 3.1). In turn, the lower conductivity in the orthogonal direction may be explained by the presence of amorphous areas, as shown in Figure 4b. These areas serve as barriers for the current applied in direction 1–1 (as shown in Figure 1a,b), as the OM images indicate that the crystalline areas located within the adjacent scanlines do not intersect (Figure 1b,c). Thus, the observed conductivity anisotropy is in good agreement with OM and Raman data. The conductivity behavior is consistent with the activation energy estimations as well. The larger conductivity is along the crystalline channels with the smaller activation energy (direction 1–2), and vice versa, the smaller conductivity and larger activation energy correspond to the orthogonal direction with the amorphous barriers (direction 1–1). The fact that the conductivity in the direction 1–1 is still almost one order of magnitude higher than that for as-deposited film is explained by the presence in this direction of only narrow amorphous stripes, separated by a highly conductive crystalline phase instead of one wide amorphous region.

An additional contribution to the conductivity in direction 1–2 may likely be caused by the laser-induced nucleation of nanocrystalline GST225 in the form of ellipsoid-shaped inclusions [29,35]. Such nanocrystals possessing form anisotropy can be represented as elongated ellipsoids, with a long axis along the applied laser field polarization [29], which coincides with the scanline direction (Figure 1b). According to the Bruggeman model for ellipsoids with such orientation [5], the conductivity should increase along the long axis, and this is in a good agreement with the obtained results. Vice versa, the contribution of LIPPS oriented in the orthogonal direction 1–1 to the conductivity anisotropy should promote decreasing conductivity along the scanlines. However, we do not see the latter effect. This is most likely explained by the following reason: the LIPPS have relatively low height, smaller than 10 nm, according to our previous study [28]. At the same time, the light penetration depth and, hence, the possible crystallization depth for as-deposited amorphous GST225 films at the applied treatment wavelength of 1250 nm are estimated to be 400 nm [44]; that is larger than the thickness of the used film (200 nm). Therefore, we can assume significant volume crystallization inside the scanlines, with dominant anisotropy axis orientation along them. When dc is applied, charge carriers interact in almost all film depths. However, the situation changes in the case of polarization-sensitive optical reflectance measurements in the near infrared range. For such measurements, we do not take into account the large-scale scanlines, as from the standpoint of an effective medium model, they do not contribute to artificial anisotropy. On the contrary, the thin LIPPS layer, which was insufficient to influence electrical conductivity, plays a dominant role of defining the main features of reflectance anisotropy in the case of optical measurements.

## 4. Conclusions

The formation of the LIPSS with the period of 1100 ± 50 nm in amorphous GST225 thin films was realized by the treatment using femtosecond laser pulses with the wavelength of 1250 nm. The orientation of such structures was orthogonal to the laser polarization, which is consistent with their formation mechanism due to SPPs generation caused by intense photoexcitation during femtosecond laser irradiation. Additionally, the structural periodicity of the irradiated samples is caused by scanlines with a large-scale period of 120 ± 10 μm. These scanlines and LIPPS are oriented mutually orthogonally. According to Raman spectroscopy, two different states of structure were observed in the irradiated area. These states correspond to alternating crystalline and amorphous areas within both the scanlines tracks and the LIPSS. This alternation was additionally confirmed via OM, where the crystalline and amorphous areas looked different due to different refractive indices.

The observed crystallization with simultaneous LIPSS formation affects both the optical and electrophysical properties of the studied samples. Reflectance spectra in the near IR range differs for different incident light polarizations. The analysis of these spectra considering interference in the thin layers and the generalized Bruggeman model allows to conclude that the presence of LIPPS provides the key contribution to optical artificial anisotropy. Moreover, the strong anisotropy of the conductivity was revealed in the temperature range of 200–400 K. The dc conductivity of the modified film is 1–5 orders of magnitude greater in the direction of the scanlines compared to both modified film conductivity in the direction orthogonal to the scanlines and the initial amorphous thin film. Such in-plane conductivity anisotropy is explained by the presence of the partially crystallized and parallel scanline tracks and the nonirradiated amorphous barriers between them, as well as the possible formation of laser-induced elongated nanocrystalline ellipsoids in the film volume. The conductivity growth after the treatment is caused by effective laser-induced crystallization, which is additionally confirmed by the activation energy estimations.

The obtained results can improve modern phase-change memory prototypes based on GST225 due to laser-induced anisotropy implementation.

## Data Availability

The data used in this research is available from the corresponding author upon reasonable request.
